# Supplementary material for: Elevated cardiovascular risk factors and chronic disease mortality in the Caribbean: A Cross-Sectional Study
Source: PLOS Glob Public Health. 2025 Sep 19;5(9):e0005209. doi: 10.1371/journal.pgph.0005209 (PMC12449024; doi:10.1371/journal.pgph.0005209)
Supplement: Attachment — Submitted filename: Response to Reviewer_PLOS.docx [file pgph.0005209.s003.docx]

**Response to Reviewers**
Manuscript ID: PGPH-D-25-00790
Title: *Elevated Cardiovascular Risk Factors and Chronic Disease Mortality in the Caribbean: A Cross-Sectional Study*

Dear Dr. Okpechi and Reviewers,

We would like to sincerely thank you for the opportunity to revise our manuscript and for the thoughtful feedback provided by the reviewers and editorial team. We appreciate the time and care taken to improve the clarity, rigor, and presentation of our study. We have carefully addressed each of the comments below and hope the revised version meets the standards of PLOS Global Public Health.

We have submitted a revised manuscript with tracked changes, an unmarked clean version, and updated supplementary material. Below we provide point-by-point responses to each comment.

**Editorial Comments**

**1. Clarify the overall aim/objectives of the study.**
Thank you for this suggestion. We revised the final paragraph of the Introduction to clearly state the overarching objectives of the study in accordance with STROBE guidelines. The revised text now reads:
*“Our objectives were to compare sex-specific and regional differences in cardiovascular mortality and risk factor profiles between the Non-Latin Caribbean and North America, and to evaluate the extent of national-level NCD policy implementation related to cardiovascular disease prevention and management.”*

**2. Clearly address issues related to differences in the imputed and complete datasets.**
We added a description of differences between the imputed and complete case datasets in the Results section, and we included a supplemental table summarizing comparisons of CVD and stroke mortality rates before and after imputation. The new supplemental table (Supplemental Table 1) presents mean differences and 95% confidence intervals to support transparency.

**3. Provide a succinct summary of key findings in the first paragraph of the Discussion.**
We revised the first paragraph of the Discussion section to summarize the three major findings of the study. The paragraph now clearly states the main results and concludes with a short interpretation. We thank the editor and reviewer for encouraging this improvement.

**Reviewer #1**

**1. Remove preliminary findings from the Introduction.**
We removed lines 54–57, which previously introduced early findings. The revised Introduction now ends with a clear statement of study objectives to better align with academic conventions.

**2. Explicitly state the study objectives.**
As described in our response to the editor above, the objectives are now clearly articulated in the final paragraph of the Introduction.

**3. Clarify rationale for excluding diabetes as a clinical risk factor.**
We appreciate this important point. In the Methods, we now note that diabetes prevalence was not included because the ENLACE dataset did not provide a diabetes-specific cardiovascular morbidity or mortality metric. We also added this as a limitation in the Discussion section:
*“Although diabetes is a known contributor to cardiovascular disease, the ENLACE dataset did not provide a cardiovascular-specific diabetes metric, limiting our ability to assess this relationship directly.”*
We cite the relevant PAHO source and acknowledge the issue raised.

**4. Provide more detail on the imputation and any post-imputation validation.**
Thank you for this thoughtful comment. The original imputation was conducted to support exploratory Pearson correlation analyses, which were ultimately excluded from the manuscript. We found that correlation testing did not meaningfully advance our primary objectives and risked cluttering the results without offering causal insight. As a result, the imputed data and associated analyses were removed. All figures and findings now reflect only available raw data, and detailed country-level values are included in Supplemental Table 1. We also clarified in the **Methods** section that although the original PAHO ENLACE dataset includes upper and lower bounds for all indicators, these were not retained in our final dataset and are not reported in this study. PAHO does not describe these bounds as formal confidence intervals, but they reflect variation in the underlying data and estimation process. We chose to emphasize direct, descriptive comparisons using harmonized country-level estimates, which we believe provide a valid and interpretable overview of regional trends.

**5. Remove interpretive language from Results.**
We removed all interpretation and recommendations from the Results section, including the specific lines cited (131–133, 142–143, 156–157, 161–162, 174–175, 193–196). Interpretations have been moved to the Discussion.

**6. Avoid “approached statistical significance” and add uncertainty measures.**
We removed all instances of the phrase “approached statistical significance.” We also added 95% confidence intervals for prevalence estimates where available and referenced this data in Supplemental Table 2.

**7. Add summary of key findings at start of Discussion.**
Addressed above in response to the editorial comment. The Discussion now begins with a succinct summary of the three main findings.

**8. Further discussion of diabetes as a risk factor.**
We briefly mention diabetes prevalence and its relevance in the Discussion but maintain that its exclusion from our dataset precluded further analysis. This is now noted explicitly as a limitation.

**9. Expand on missing data as a limitation.**
Resolved in 4.

**Reviewer #2**

**1. Move lines 54–57 from the Introduction to the Discussion.**
Completed. These lines were removed entirely from the Introduction, and relevant content is now part of the revised opening paragraph of the Discussion.

**2. Describe countries’ geography, population, and health indicators.**
We added a sentence in the Methods under “Data Sources” to provide brief geographic and demographic context for the Non-Latin Caribbean region. However, we intentionally kept this concise to avoid repetition, since specific country-level indicators are detailed in the Results.

**Journal Requirement**

**1. Remove “data not shown.”**
We removed all instances of “data not shown.”

**Closing remarks**

We hope these revisions meet the expectations of the reviewers and editorial team. We thank you again for your time, effort, and constructive feedback, and we look forward to the opportunity to contribute to the important work published in PLOS Global Public Health.

Sincerely,
Cesar Barrabi, PhD
On behalf of all authors
